# Rare Complication of a Rare Malignancy: Case Report of Cardiac Amyloidosis Secondary to Waldenstrom Macroglobulinemia

**DOI:** 10.5339/qmj.2022.7

**Published:** 2022-03-22

**Authors:** Aaron Charles Lobo, Vivek Bhat, Seetharam Anandram, Shanthala Devi A.M, Sanjukta S. Rao, Ge-vivin Vinister, Veronica Lobo, Cecil Reuben Ross

**Affiliations:** ^1^Department of General Medicine & Hematology, St. John's Medical College, Bangalore, India E-mail: loboaaron@gmail.com; ^2^Faculty of Medicine, St. John's Medical College, Bangalore, India; ^3^Department of Transfusion Medicine and Immunohematology, St. John's Medical College, Bangalore, India

**Keywords:** heart failure, restrictive cardiomyopathy, monoclonal gammopathy, amyloidosis, chemotherapy

## Abstract

Cardiac amyloidosis is a rare disorder caused by the myocardial deposition of abnormal fibrils. A 52-year-old man was referred to our center with clinical features of heart failure, after cardiac magnetic resonance imaging showed restrictive cardiomyopathy. Abdominal fat pad biopsy showed features of amyloidosis, and after hematological workup, he was diagnosed with Waldenstrom macroglobulinemia (WM). He was initiated on a rituximab-based chemotherapy regimen, and his cardiac function was assessed serially. Because of non-response, he was switched to a bortezomib-based regimen. Unfortunately, three days into this regimen, the patient died. WM is a rare plasma cell dyscrasia with a nonspecific presentation. It uncommonly presents with sequelae of amyloidosis–the IgM subtype of amyloid-light chain (AL) amyloidosis. Diagnostic delays are common, contributing to an already poor prognosis. Amyloidosis in WM requires urgent treatment – clonal chemotherapy, and supportive cardiac care in heart involvement. Bortezomib-based regimens are commonly recommended, with diuretics as the mainstay for cardiac treatment. However, in most advanced cases, the prognosis is poor; thus, a high degree of suspicion is necessary for early diagnosis. This case illustrates the possible presentation of cardiac amyloidosis as a rare malignancy.

## Introduction

Amyloidosis is a rare condition wherein the extracellular deposition of abnormal fibrillar proteins causes organ dysfunction. Amyloid-light chain (AL) amyloidosis, where the fibrillar proteins are derived from immunoglobulin light chains, is the most common type.^1^ Cardiac amyloidosis results in restrictive cardiomyopathy, manifesting as heart failure or arrhythmias.^1,2^ It is often diagnosed late, with a fatal outcome.^3^

Waldenstrom macroglobulinemia (WM) is a rare plasma cell dyscrasia, which uncommonly presents with amyloidosis. AL amyloidosis due to the deposition of IgG light chains, as in multiple myeloma, is well known, whereas IgM-related amyloidosis, as seen in WM, is rarely reported. This report adds to the available literature the case of a patient who presented with heart failure because of cardiac amyloidosis secondary to WM and advocates for a greater degree of suspicion for these conditions.

## Case Report

A 52-year-old man with well-controlled type 2 diabetes mellitus and hypertension, for the past five years, and without a history of smoking or alcohol use, presented with features of exertional breathlessness that progressed from class 2 to class 4 according to the New York Heart Association functional classification over six months. After much delay since the onset of his symptoms, he was diagnosed with restrictive cardiomyopathy based on cardiac magnetic resonance imaging and echocardiography and was referred to our center after abdominal pad fat biopsy showed features of extracellular proteinaceous deposits that were positive in Congo red staining, consistent with amyloidosis.

**Figure 1.**
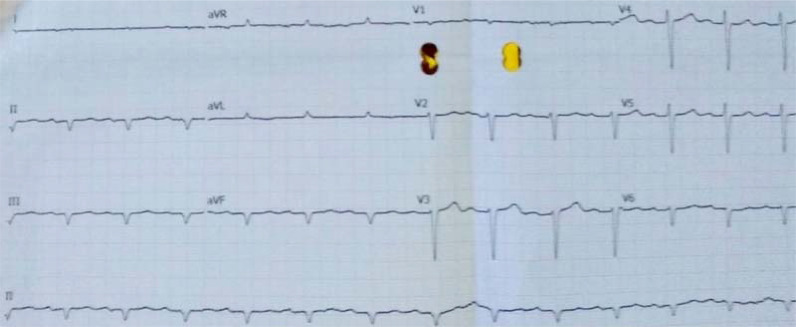
Electrocardiogram showing low-voltage QRS complexes in limb leads, prolonged PR interval with type 1 atrioventricular block, left axis deviation, Q waves in inferior leads, and poor R wave progression.

**Figure 2.**
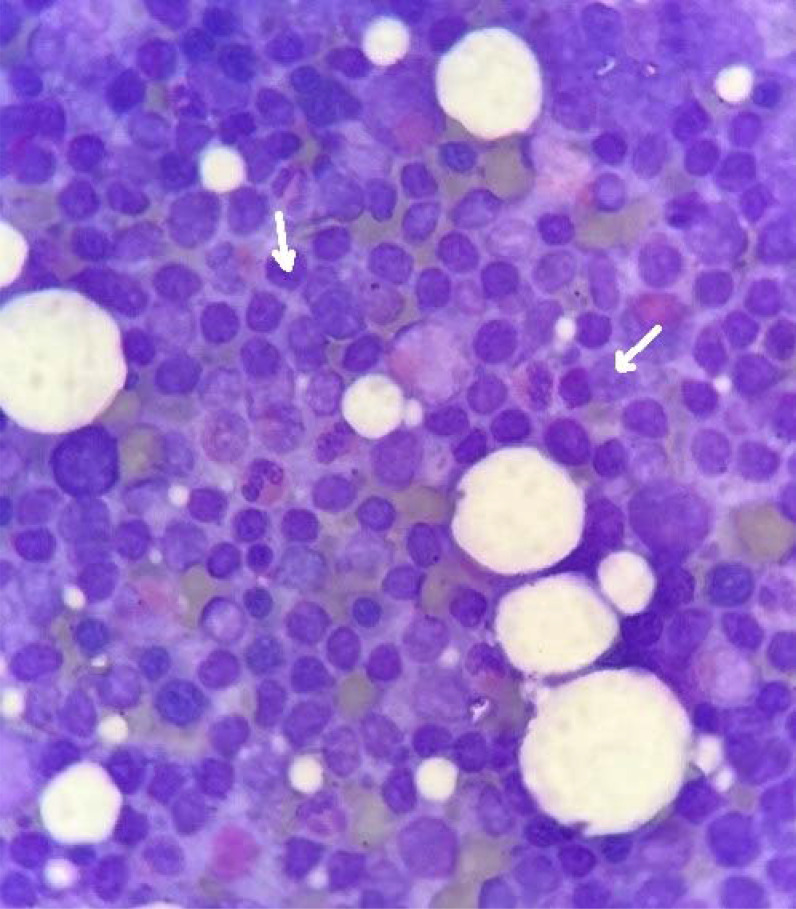
Imprint smear of the bone marrow showing increased lymphocytes (white arrows), suggestive of lymphoplasmacytic lymphoma.

**Figure 3.**
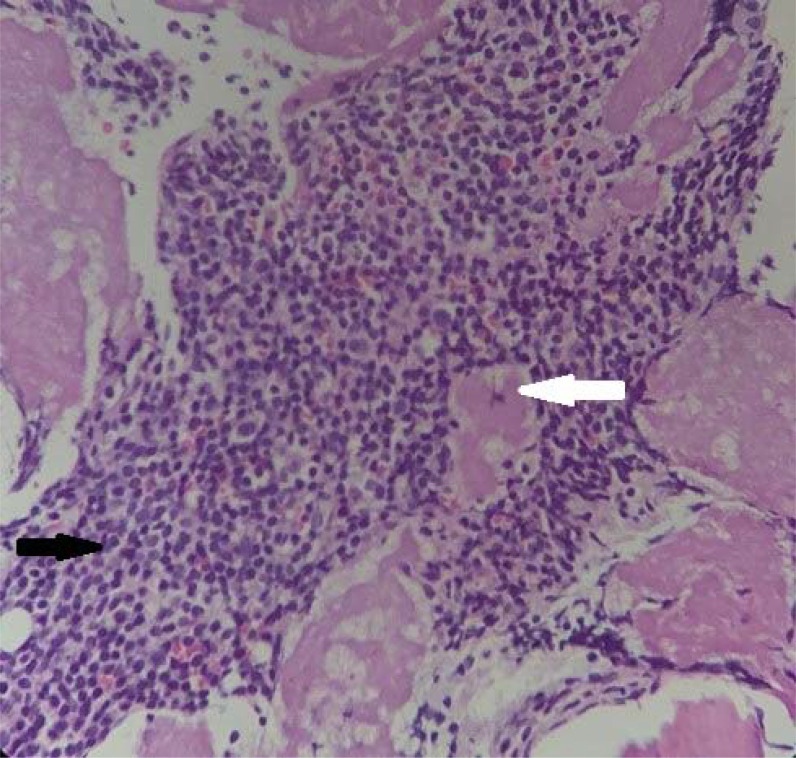
Trephine biopsy showing sheets of lymphocytes (rightward black arrow) and extracellular homogenous eosinophilic deposition (leftward white arrow), suggestive of amyloidosis.

At his first admission, clinical examination revealed pedal edema up to the mid-shin, elevated jugular venous pressure, and minimal basal lung crepitations. Heart sounds were normal. He had no pallor, lymphadenopathy, palpable hepatosplenomegaly, macroglossia, ecchymosis, orthostatic hypotension, or evidence of peripheral neuropathy. His blood counts revealed hemoglobin of 11.7 g/dL, leucocyte count of 6840 cells/mm^3^, and platelet count of 167,000 cells/mm^3^. The peripheral smear was normal. Stool occult blood was negative, and levels of serum ferritin, vitamin B12, and folate were all normal. His liver function tests were largely within the normal limits (Table 1). His serum creatinine and urea levels were 1.23 mg/dL and 37 mg/dL, respectively. Urinalysis did not show proteinuria or glycosuria. Abdominal ultrasonography revealed mild hepatosplenomegaly. Electrocardiogram (ECG) showed low-voltage QRS complexes in limb leads, prolonged PR interval with type 1 atrioventricular block, left axis deviation, Q waves in inferior leads, and poor R wave progression (Figure 1). Echocardiography showed concentric left ventricular (LV) hypertrophy with global hypokinesia, diastolic dysfunction, and an ejection fraction of 30%. The free light chain ratio (kappa: lambda) was 15.35 (218:14.2), with a difference in free light chain (dFLC) of 203.8 mg/dL. There was an M-band in the β2 globin region (1.4 g/dL) on serum protein electrophoresis, and subsequent serum immunofixation analysis revealed features of an IgM paraproteinemia. His imprint smears showed increased lymphocytes (Figure 2), and bone marrow biopsy showed sheets of lymphocytes with extracellular homogenous eosinophilic deposition (Figure 3). Immunohistochemistry showed leukocyte common antigen (LCA), CD79a, CD20, and CD138 positivity. A final diagnosis of WM with cardiac amyloidosis was made.

He was initiated on a regimen of rituximab, cyclophosphamide, and dexamethasone given every four weeks–on day 1, 20 mg dexamethasone and 375 mg/m^2^ rituximab given intravenously, along with cyclophosphamide 100 mg/m^2^ administered orally twice a day, followed by cyclophosphamide alone on days 2–5. Before chemotherapy initiation, his troponin I level was 0.057 ng/dL, and the NT-proBNP was 5020 pg/mL. His cardiac function was assessed with serial echocardiograms, troponin I, and NT-proBNP levels. Over the subsequent two cycles of chemotherapy, there was a progressive worsening of his symptoms, signs, and laboratory parameters despite maximum tolerated doses of furosemide, spironolactone, and carvedilol. After the first cycle, his troponin I and NT-proBNP levels were 0.040 ng/dL and 5400 pg/mL, respectively. His NT-proBNP further increased to 9820 pg/mL after the second cycle. Because of non-improvement, the regimen was changed at the start of the third cycle to one consisting of bortezomib, rituximab, and dexamethasone. At this point, his troponin I and NT-proBNP levels were 0.462 ng/mL and 11500pg/mL, respectively, with an ejection fraction of 25%, and end-diastolic LV thickness of 40 mm. His last measured hematologic and biochemical parameters are summarized in Table 1. Three days into this regimen, his cardiac function worsened suddenly over a few hours. On examination, he was hypotensive and had bradycardia. ECG showed sinus bradycardia (heart rate of 40 beats per minute), multiple ventricular premature complexes, and Q waves in inferior leads. He was transferred to the coronary care unit and initiated on inotropic support. The repeat echocardiography did not show any motion-wall abnormalities, and troponin I levels were unchanged. A temporary pacemaker line was inserted. The patient developed asystole, and despite multiple cycles of cardiopulmonary resuscitation, he could not be revived.

## Discussion

WM is a rare lymphoplasmacytic lymphoma with monoclonal IgM paraprotein.^4^ Many patients are asymptomatic, and among those with symptoms, the presentation is nonspecific, with weakness, anorexia, and weight loss.^5^ Physical examination may reveal signs of anemia, peripheral neuropathy, hepatosplenomegaly, and lymphadenopathy. These were not present in our patient, and he presented only after developing sequelae of amyloidosis. Definitive diagnosis requires a bone marrow examination demonstrating infiltration by clonal lymphoplasmacytic cells and monoclonal IgM.^5^

Less than 10% of patients with WM have amyloidosis.^6^ To our knowledge, this is only the second such case reported from India.^7^ While AL amyloidosis is well recognized in IgG gammopathies, such as multiple myeloma, IgM-related AL amyloidosis, as seen in WM, is much more uncommon. Given the nonspecific presentation, these patients often present late in the course of disease^3^; at which point, the prognosis is dismal.

AL amyloidosis is the most common type of systemic amyloidosis, where the amyloid fibrils are derived from monoclonal free light chains. While Congo red staining can provide histological confirmation of amyloidosis, the subtype can only be confirmed by fibrillary typing methods such as mass spectrometry.^8^ Cardiac involvement is seen in approximately half of these patients, manifesting most commonly as heart failure, with other manifestations including cardiac autonomic neuropathy and arrhythmias.^1,2^ Cardiac involvement in patients with WM having amyloidosis has been shown to confer further poor prognostication.^6^ This, along with the late stage at which our patient presented, probably contributed to his demise, despite appropriate chemotherapy.

Indications for treatment in WM include B symptoms, symptomatic organomegaly, and/or lymphadenopathy, with amyloidosis representing an urgent indication.^9^ Rapid reduction in IgM levels is required, and the preferred regimen is bortezomib with rituximab (BR) or BR with dexamethasone (BDR).^9^ However, primary treatment with rituximab-based regimens has shown promising results, with our regimen of dexamethasone–rituximab–cyclophosphamide having a major response rate of 74%, and low toxicity.^4,10^ A change in therapy is warranted after two to three cycles if amyloidogenic precursors do not reduce.^2^ We started our patient on a rituximab-based regimen to minimize the risk of arrhythmias because of bortezomib; due to non-improvement, this regimen was switched. However, patients with IgM-associated amyloidosis often still do not respond.^11^

In cardiac amyloidosis, therapy aims to halt abnormal protein production and provide supportive measures with volume control using diuretics. Arrhythmias need appropriate treatment. Those without significant extracardiac involvement may benefit from a heart transplant.^1^

Age, hemoglobin, platelet count, β2 microglobulin, and IgM concentrations are traditional prognostic indicators in WM, but in those with amyloidosis, cardiac involvement, most sensitively indicated by NT-pro BNP drives the prognosis.^1,11^

Therefore, cardiac amyloidosis must be considered in a patient with cardiac symptoms without a previous history of myocardial ischemia, those with arrhythmias in addition to increased ventricular wall thickness, and those with elevated biomarkers without significant findings on coronary angiography, as in our case. Our case highlights the possible presentation of WM, an extremely rare condition, as cardiac amyloidosis. Clinicians must have a high degree of suspicion and rapidly initiate treatment.

## Figures and Tables

**Table 1:** Basic biochemical and hematologic parameters during patient’s hospitalization

Investigative value	At admission	Last measured	Normal range

Hemoglobin	11.7 g/dL	13.2 g/dL	12–16 g/dL

Total leukocyte count	6840/mm^3^	6240/mm^3^	4000–11000/mm^3^

Platelet count	167000/mm^3^	75000/mm^3^	150000–400000/mm^3^

Serum protein	7.4 g/dL	7.56 g/dL	6.4–8.3 g/dL

Serum albumin	3.3 g/dL	3.1 g/dL	2.9–4.5 g/dL

Total bilirubin	0.99 mg/dL	5.12 mg/dL	0.2–1.2 mg/dL

Direct bilirubin	0.42 mg/dL	2.51 mg/dL	0.0–0.5 mg/dL

Alanine transaminase	40 U/L	419 U/L	5–34 U/L

Aspartate transaminase	28 U/L	319 U/L	5–34 U/L

Alkaline phosphatase	143 U/L	119 U/L	48–95 U/L

Gamma glutamyl transferase	96 U/L	109 U/L	9–36 U/L

Serum creatinine	1.23 mg/dL	1.55 mg/dL	0.72–1.25 mg/dL

Serum urea	37 mg/dL	97 mg/dL	19–44 mg/dL


